# Rural general practitioners have different personal and professional trajectories from those of their urban colleagues: a case-control study

**DOI:** 10.1186/s12909-023-04794-0

**Published:** 2023-11-07

**Authors:** Perrine Nedelec, Laurélie Beviere, Anthony Chapron, Maxime Esvan, Julien Poimboeuf

**Affiliations:** 1 Department of General Practice, Univ Rennes, 35000 Rennes, France; 2 Department of General Practice, Univ Rennes, CHU Rennes, 35000 Rennes, France; 3 INSERM, CIC-1414, Primary Care Research Team, F-35000 Rennes, France

**Keywords:** Case–control study, General Practitioner, Rural environment, Rural origin

## Abstract

**Background:**

In France, rural general practitioner (GP) numbers could reduce by 20% between 2006 and 2030 if no measures are taken to address primary care access difficulties. In countries such as Australia, the USA and Canada, intrinsic and extrinsic factors associated with GPs practising in rural areas include rural upbringing and rural training placements. However, the health systems and rural area definition differ between these countries and France making result extrapolation difficult. These factors must be studied in the context of the French heath system, to design strategies to improve rural GP recruitment and retention. This study aims to identify the intrinsic and extrinsic factors associated with GPs practising in rural areas in France.

**Methods:**

This case–control study was conducted between May and September 2020. Included GPs practised in Brittany, France, and completed a self-administered questionnaire. The cases were rural GPs and controls were urban GPs. National references defined rural and urban areas. Comparisons between rural and urban groups were conducted using univariate and multivariate analyses to identify factors associated with practising in a rural area.

**Results:**

The study included 341 GPs, of which 146 were in the rural group and 195 in the urban group. Working as a rural GP was significantly associated with having a rural upbringing (OR = 2.35; 95% CI [1.07–5.15]; p = 0.032), completing at least one undergraduate general medicine training placement in a rural area (OR = 3.44; 95% CI [1.18–9.98]; *p* < 0.023), and having worked as a locum in a rural area for at least three months (OR = 3.76; 95% CI [2.28–6.18]; *p* < 0.001). Choosing to work in a rural area was also associated with the place of residence at the end of postgraduate training (OR = 5.13; 95% CI [1.38–19.06]; p = 0.015) and with the spouse or partner having a rural upbringing (OR = 2.36; 95% CI [1.12–4.96]; p = 0.023) or working in a rural area (OR = 5.29; 95% CI [2,02–13.87]; *p* < 0.001).

**Conclusions:**

French rural GPs were more likely to have grown up, trained, or worked as a locum in a rural area. Strategies to improve rural GP retention and recruitment in France could therefore include making rural areas a more attractive place to live and work, encouraging rural locum placements and compulsory rural training, and possibly enrolling more medical students with a rural background.

## Introduction

Access to primary care is a major public health issue in many countries throughout the world, including Europe, and is accentuated in rural areas [1, 2]. However, the causes for this problem vary between countries. For example, in Australia, the GP population is ageing and there are vast distances between regional cities [3]. Whereas in Canada, only 8.5% of GPs work in rural areas but care for 18% of the population [4]. In France, rural GP numbers are decreasing and are expected to reduce by 20% between 2006 and 2030, if no measures are taken to address primary care access difficulties [5]. This is a major public health problem in France since rural areas have the largest proportion of the French population with the lowest accessibility to GPs [6].

To date, little is known about the intrinsic and extrinsic factors affecting rural GP recruitment and retention in France. However, substantial evidence exists in countries such as Australia, Canada, and the United Kingdom to indicate that social and environmental factors appear to have a greater influence on where GPs choose to practise than financial or material factors [7, 8]. GPs having a rural upbringing is the intrinsic factor most likely to influence a GP to practise in a rural area, according to a 2020 literature review including Australia, Canada, and the United Kingdom. Other intrinsic factors included being in a stable relationship, having pre-school children or children in primary school, and being interested in rural medical practise even before starting medical school. Extrinsic factors included completing rural undergraduate and postgraduate training placements [9].

These intrinsic and extrinsic factors could be considered when developing strategies to improve rural GP recruitment and retention. For example, selectively recruiting students identified as being the most likely to practise in rural areas and rural exposure during undergraduate training [10, 11]. However, none of these strategies are currently used in France. Several financially-based incentives to encourage GPs to practise in GP shortage areas in France have been implemented but have only been moderately effective as they did not consider social aspects and only created an opportunity effect [7, 12].

Currently, most research into factors influencing where GPs choose to practise has been conducted in Canada, Australia, and the USA but little comparable data is available for European countries. Furthermore, the health systems and the definition of a rural area in these countries differ from France where rural areas are more densely populated. This makes extrapolating results from these geographically different countries to European countries such as France extremely difficult. Establishing what factors influence where GPs choose to practise in France will help to determine which strategies could effectively improve rural GP recruitment and retention.

This study therefore aims to identify the intrinsic and extrinsic factors associated with GPs practising in rural areas in France.

## Methods

This case–control study was conducted between May and September 2020 among GPs from Brittany, France. The rural group contained GPs working in rural areas and the control group contained GPs working in urban areas. Communes were classified as rural or urban using the national reference coding system (INSEE 2010) [13]. This system is based on the number of inhabitants in the commune, the continuity of built-up areas and the influence of neighbouring towns and cities.

A complete list of GPs in Brittany, western France region, in 2019 was obtained from the national GP register. GPs working in a private practice in Brittany were included. GPs who were no longer working, retired, working as a locum or whose main activity was not general practice were excluded.

The number of participants required was calculated based on English-language literature [14, 15]. Using this literature, it was assumed that 37% of GPs working in rural practice grew up in a rural area, compared with 22% who work in an urban practice. An alpha risk of 0.05 and a power of 80% was used revealing that 144 participants per group were required.

The self-administered study questionnaire was designed using Limesurvey® software and was based on French and international literature. It was divided into six sections: sociodemographic data, primary and secondary school education, undergraduate training, postgraduate training, locum work and practising as a GP. The primary endpoint was to compare the proportion of GPs with a rural upbringing in the rural and urban groups. Volunteer GPs tried the questionnaire to ensure all questions could be understood. Their mean response time was 5 min. Questionnaires were sent by e-mail whenever possible and by post in the absence of an e-mail address. When sent by post, an explanatory cover letter and a stamped return envelope were sent with the questionnaire. A first reminder was sent to the rural GP group by phone or e-mail, and then a second reminder by phone only. Each non-respondent was called in the random order obtained from the selection process. No reminders were sent to the urban GP group. All the questionnaires were anonymised. Data from each questionnaire were entered on an Excel spreadsheet. Any questions requiring the commune name were re-classified in a second step according to their INSEE zone [13]. GP participation was voluntary and no renumeration was given.

### Statistical analysis

A descriptive data analysis was performed. Numbers and percentages were calculated for qualitative variables and means, standard deviations, quartiles, and minimum and maximum values were calculated for quantitative variables. The normality of the quantitative variable distributions was checked. The different variables were compared between groups using Student's t-test for quantitative variables, and Chi^2^ tests or Fisher’s exact tests for qualitative variables. Comparisons between the rural and urban groups were conducted using univariate and multivariate analyses to identify factors associated with practising in a rural area. All statistical tests had a significance threshold of 0.05. The statistical analyses were conducted using SAS software v.9.4® (SAS Institute, Cary, NC. USA).

## Results

### Descriptive analyses

#### Sociodemographic data

**Fig. 1:**
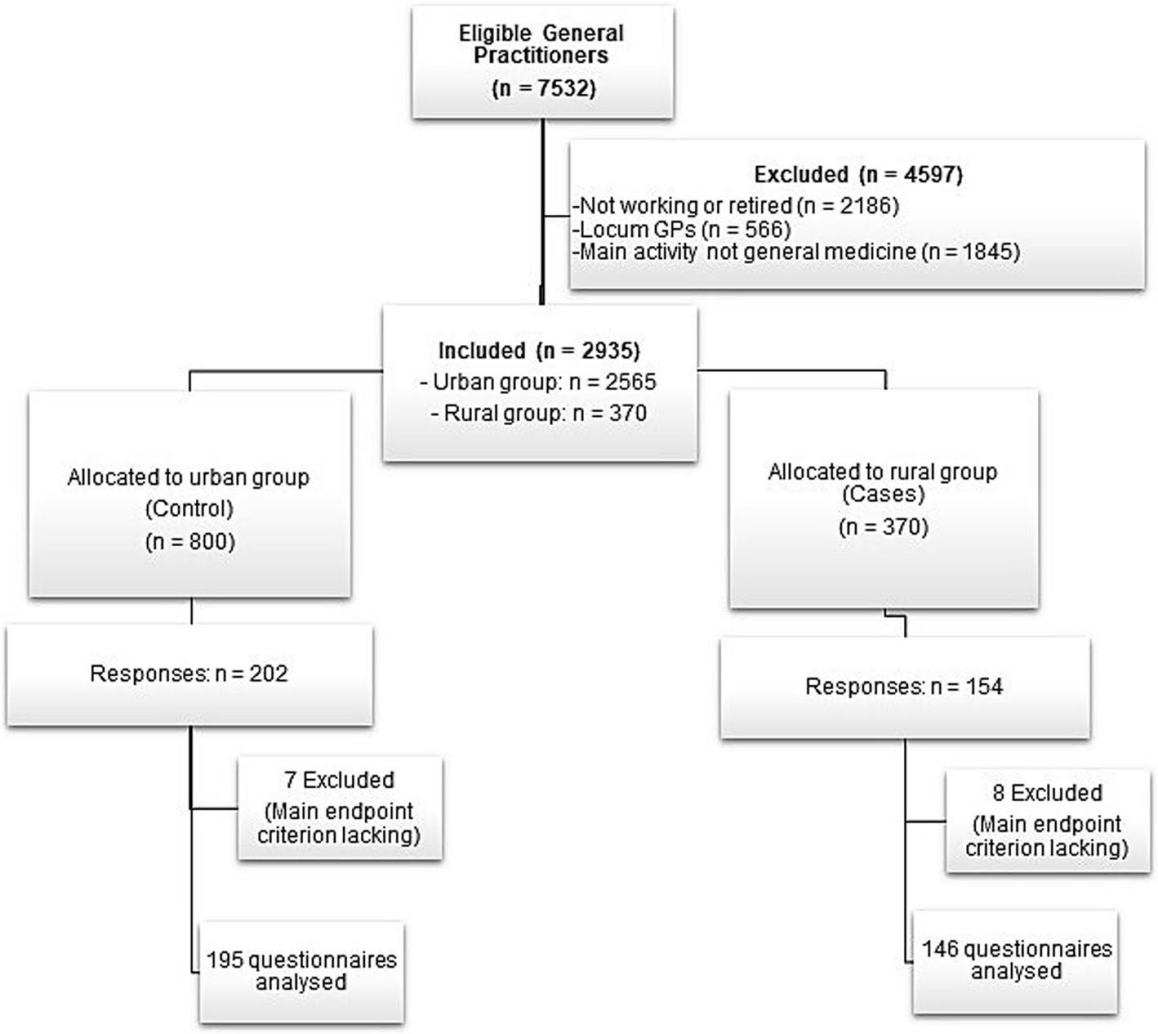
Flow-chart showing numbers of participants at each stage

Of the 7532 GPs registered, 4597 were excluded, of which 2186 were not practising, 566 were locum GPs, 1424 were not in private practice and 421 had a main activity that was not general practice. In total, 2935 GPs met the inclusion criteria. They were grouped into rural (370 GPs) and urban (2565 GPs) according to where they practised (Fig. 1). To account for non-responses and unusable questionnaires, all 370 rural GPs were approached, and 800 urban GPs were selected using simple random selection. In total, 341 GPs were included, of which 195 GPs were included in the urban group and 146 in the rural group. GP characteristics are described in Table 1. The mean age of respondents in the total study population was 49.2 years and 51.6% (*n* = 176) were women, with no significant difference between groups for these characteristics (*p* = 0.150 and *p* = 0.938 respectively). There was a significant difference between where GPs lived with 59.6% of the rural GPs living in rural areas versus just 2.1% of urban GPs (*p* < 0.001).

**Table 1:** Sociodemographic data of participating general practitioners

Variable	Urban general practitioners *n* = 195	Rural general practitioners *n* = 146	*p-value*
**Age**^b^			*p* = *0.150 (S)*
	48.4 ± 11.7	50.3 ± 12.5	
**Gender**^a^			*p* = *0.938 (C)*
Male	94 (48.2%)	71 (48.6%)	
Female	101 (51.8%)	75 (51.4%)	
**Commune of present practice **^**a**^			*p* < *0.001* *(F)*
Large urban area	87 (44.6%)	0 (0.0%)	
Periphery of urban areas	71 (36.4%)	0 (0.0%)	
Multipolar communes in large urban areas	18 (9.2%)	0 (0.0%)	
Medium sized urban centres	16 (8.2%)	0 (0.0%)	
Small centres	3 (1.5%)	0 (0.0%)	
Other multipolar communes	0 (0.0%)	90 (61.6%)	
Communes distant from urban influence	0 (0.0%)	56 (38.4%)	
**Present place of residence**^a^			*p* < *0.001* *(C)*
Urban	191 (97.9%)	59 (40.4%)	
Rural	4 (2.1%)	87 (59.6%)	
**Date of starting in present practice**^a^			*p* = *0.428* *(F)*
1960 to 1980	4 (2.1%)	6 (4.1%)	
1981 to 1990	29 (14.9%)	24 (16,4%)	
1991 to 2000	44 (22.6%)	23 (15.8%)	
2001 to 2010	35 (17.9%)	24 (16.4%)	
2011 to 2020	83 (42.6%)	69 (47.3%)	

(S) Student's t-test

(C) Chi^2^ tests

(F) Fisher’s exact tests

^a^Qualitative variables: number (%)

^b^Quantitative variables: mean ± SD

#### Personal and family characteristics

A significant difference was observed between the origins of the two groups (*p* = 0.02) with 12.3% of rural GPs having a rural upbringing compared with 5.6% of urban GPs. Also, significantly more rural GPs (8.1%) lived in a rural area during the last term of postgraduate training than urban GPs 1.7% (*p* = 0.007). No significant differences were found between the groups for the mother's profession (*p* = 0.525), the father's profession (*p* = 0.560) and leisure activities (*p* = 0.903) (Table 2).

**Table 2:** Personal and family characteristics of participating GPs

Variable	Urban practitioners *n* = 195	Rural practitioners *n* = 146	*p-value*
**Place of origin**^a^			*p* = *0.029* *(C)*
Urban	184 (94.4%)	128 (87.7%)	
Rural	11 (5.6%)	18 (12.3%)	
**Mother's socio-professional category**^a^			*p* = *0.525* *(F)*
Farming	7 (3.6%)	9 (6.2%)	
Self-employed, trade, small business	6 (3.1%)	6 (4.1%)	
Professional	30 (15.4%)	26 (17.8%)	
Intermediate	46 (23.6%)	27 (18.5%)	
Salaried worker	31 (15.9%)	29 (19.9%)	
Manual worker	0 (0.0%)	1 (0.7%)	
Retired	1 (0.5%)	0 (0.0%)	
Unemployed	74 (37.9%)	48 (32.9%)	
**Father's socio-professional category**^a^	***n* = 191**	***n* = 145**	*p* = *0.560* *(F)*
Farming	7 (3.7%)	11 (7.6%)	
Self-employed, trade, small business	18 (9.4%)	16 (11.0%)	
Professional	96 (50.3%)	63 (43.4%)	
Intermediate	33 (17.3%)	30 (20.7%)	
Salaried worker	18 (9.4%)	14 (9.7%)	
Manual worker	17 (8.9%)	9 (6.2%)	
Retired	0 (0.0%)	0 (0.0%)	
Unemployed	2 (1.0%)	2 (1.4%)	
**Marital status at the start of practise**^a^	***n* = 191**	***n* = 134**	*p* = *0.559* *(C)*
Married or with a partner	167 (87.4%)	120 (89.6%)	
Single	24 (12.6%)	14 (10.4%)	
**If married or with a partner, spouse or partner from a rural area **^**a**^	***n* = 165**	***n* = 119**	*p* = *0.021* *(C)*
Yes	13 (7.9%)	20 (16.8%)	
No	152 (92.1%)	99 (83.2%)	
**If married or with a partner, socio-professional category of spouse or partner **^**a**^	***n* = 167**	***n* = 120**	*p* = *0.283* *(F)*
Farming	3 (1.8%)	1 (0.8%)	
Self-employed, trade, small business	5 (3.0%)	3 (2.5%)	
Professional	81 (48.5%)	48 (40.0%)	
Intermediate	48 (28.7%)	31 (25.8%)	
Salaried worker	10 (6.0%)	12 (10.0%)	
Manual worker	1 (0.6%)	3 (2.5%)	
Retired	0 (0.0%)	1 (0.8%)	
Unemployed	19 (11,4%)	21 (17.5%)	
**If married or with a partner, area where the spouse or partner is working**^a^	***n* = 149**	***n* = 99**	*p* < *0.001* *(C)*
Urban	143 (96.0%)	81 (81.8%)	
Rural	6 (4.0%)	18 (18.2%)	
**Number of children when started at current practice**^a^	***n* = 191**	***n* = 134**	*p* = *0.204 (F)*
0	51 (26.7%)	45 (33.6%)	
1	47 (24.6%)	38 (28.4%)	
2	64 (33.5%)	33 (24.6%)	
3	21 (11.0%)	15 (11.2%)	
4	8 (4.2%)	2 (1.5%)	
5 or more	0 (0.0%)	1 (0.7%)	
**Leisure activity when started at current practice**^a^	***n* = 191**	***n* = 134**	*p* = *0.903 (C)*
Yes	70 (36.6%)	50 (37.3%)	
No	121 (63.4%)	84 (62.7%)	
**Rural area of residence at the end of postgraduate training**^a^	***n* = 177**	***n* = 123**	*p* = *0.007 (C)*
Yes	3 (1.7%)	10 (8.1%)	
No	174 (98.3%)	113 (91.9%)	

(S) Student's t-test

(C) Chi^2^ tests

(F) Fisher’s exact tests

^a^Qualitative variables: number (%)

Furthermore, significantly more rural GPs had a spouse or partner with a rural upbringing (*p* = 0.021) and who were already working in a rural area when the GP started working at their current practice, (18.2%) compared with those of urban GPs (4%) (*p* < 0.001). No significant difference was noted for the socio-professional category of the spouses or partners (*p* = 0.283) (Table 2).

#### University and professional careers

Rural GPs were significantly more likely to have completed at least one rural undergraduate training placement (8.3% vs. 2.6%) (*p* = 0.017). Rural GPs were also more likely to have had a level 1 (supervised) placement (23.2% vs. 14.4%) (*p* = 0.051) or a level 2 (non-supervised) placement (16.0% vs. 9.4%) (*p* = 0.081) in a rural area during their postgraduate training but the difference was not significant. The same trend was observed when the two placement types were pooled (*p* = 0.066). Furthermore, rural GPs were significantly more likely to have been a locum for longer than three months in a rural area (45.6% vs. 18.2%) (*p* < 0.001) (Table 3).

**Table 3:** University and professional characteristics of participating GPs

Variable	Urban practitioners *n* = 195	Rural practitioners *n* = 146	*p-value*
**Aiming for general medicine when enrolled **^**a**^	***n* = 195**	***n* = 146**	*p* = *0.200* *(C)*
Yes	76 (39.0%)	67 (45.9%)	
No	119 (61.0%)	79 (54.1%)	
**General medicine placement during undergraduate years **^**a**^	***n* = 195**	***n* = 145**	*p* = *0.322* *(C)*
Yes	83 (42.6%)	54 (37.2%)	
No	112 (57.4%)	91 (62,8%)	
**Rural general medicine undergraduate placement **^**a**^	***n* = 194**	***n* = 144**	*p* = *0.017* *(C)*
Yes	5 (2.6%)	12 (8.3%)	
No	189 (97.4%)	132 (91.7%)	
**Medical speciality targeted in 6**^**th**^** year **^**a**^	***n* = 195**	***n* = 145**	*p* = *0.129* *(F)*
General medicine	141 (72.3%)	118 (81.4%)	
Other speciality	28 (14.4%)	14 (9.7%)	
Surgical speciality	6 (3.1%)	4 (2.8%)	
Did not know	14 (7.2%)	9 (6.2%)	
Other	6 (3.1%)	0 (0.0%)	
**Postgraduate placement **^**a**^	***n* = 194**	***n* = 139**	*p* = *0.266* *(C)*
Yes	181 (93.3%)	125 (89.9%)	
No	13 (6.7%)	14 (10.1%)	
**Postgraduate general medicine placement **^**a**^	***n* = 194**	***n* = 139**	*p* = *0.946* *(C)*
Yes	143 (73.7%)	102 (73.4%)	
No	51 (26.3%)	37 (26.6%)	
**If placement in general medicine, level 1 (supervised) **^**a**^	***n* = 130**	***n* = 88**	*p* = *0.017* *(C)*
Yes	116 (89.2%)	68 (77.3%)	
No	14 (10.8%)	20 (22.7%)	
**Rural level 1 general medicine placement **^**a**^	***n* = 180**	***n* = 125**	*p* = *0.051* *(C)*
Yes	26 (14.4%)	29 (23.2%)	
No	154 (85.6%)	96 (76.8%)	
**If placement in general medicine, level 2 (unsupervised) **^**a**^	***n* = 130**	***n* = 88**	*p* = *0.584* *(C)*
Yes	64 (49.2%)	40 (45.5%)	
No	66 (50.8%)	48 (54.5%)	
**Rural level 2 general medicine placement **^**a**^	***n* = 181**	***n* = 125**	*p* = *0.081* *(C)*
Yes	17 (9.4%)	20 (16.0%)	
No	164 (90.6%)	105 (84.0%)	
**Level 1 or 2 general medicine placement in rural area**	***n* = 180**	***n* = 125**	*p* = *0.066* *(C)*
Yes	33 (18.3%)	34 (27.2%)	
No	147 (81.7%)	91 (72.8%)	
**Locum for more than 3 months **^**a**^	***n* = 194**	***n* = 137**	*p* = *0.137* *(C)*
Yes	150 (77.3%)	96 (70.1%)	
No	44 (22.7%)	41 (29.9%)	
**Locum for more than 3 months in rural area **^**a**^	***n* = 192**	***n* = 136**	*p* < *0.001* *(F)*
Yes	35 (18.2%)	62 (45.6%)	
No	157 (81.8%)	74 (54.4%)	

(C) Chi^2^ tests

(F) Fisher’s exact tests

^a^Qualitative variables: number (%)

#### Factors associated with choosing to practise in a rural area

Univariate analysis revealed several factors that are associated with a GP choosing to practise in a rural area (Table 4). These include having a rural upbringing (OR = 2.35; 95% CI [1.07–5.15]; *p* = 0.032), completing at least one rural undergraduate general medicine training placement (OR = 3.44; 95% CI [1.18–9.98]; *p* = 0.023), living in a rural area during the last six months of postgraduate training (OR = 5.13; 95% CI [1.38–19.06]; *p* = 0.015), working as a locum in a rural area for at least three months (OR = 3.76; 95% CI [2.28–6.18]; *p* < 0.001), and having a spouse or partner with a rural upbringing (OR = 2.36; 95% CI [1.12–4.96]; *p* = 0.023) or working in a rural area (OR = 5.29; 95% CI [2,02–13.87]; *p* < 0.001).

**Table 4:** Factors associated with practising in a rural area: univariate analyses

Variable	Number of respondents	Number of rural GPs	Odds ratio [95%CI]	*p-value*
**Place of origin**^**a**^	341	146		*p* = *0.032*
Urban	312	128	1	
Rural	29	18	2.35 [1.07—5.15]	
**Marital status at start of practise**	325	134		*p* = *0.559*
Single	38	14	1	
With a partner	287	120	1.23 [0.61—2.48]	
**If married or with a partner, childhood residence of spouse or partner in rural area**	284	119		*p* = *0.023*
No	251	99	1	
Yes	33	20	2.36 [1.12—4.96]	
**If married or with a partner, his/her workplace**	248	99		*p* < *0.001*
Urban	224	81	1	
Rural	24	18	5.29 [2.02—13.87]	
**Children when started at current practice**	325	134		*p* = *0.182*
No	96	45	1	
Yes	229	89	0.72 [045—1.17]	
**Rural undergraduate general medicine placement**	338	144		*p* = *0.023*
No	321	132	1	
Yes	17	12	3.44 [118—9.98]	
**Postgraduate placement**	333	139		*p* = *0.270*
No	27	14	1	
Yes	306	125	0.64 [0.29—1.41]	
**Postgraduate general medicine placement**	333	139		*p* = *0.946*
No	88	37	1	
Yes	245	102	0.98 [0.60—161]	
**Rural level 1 (supervised) postgraduate general medicine placement**	305	125		*p* = *0.052*
No	250	96	1	
Yes	55	29	1.79 [0.99 – 3.22]	
**Rural level 2 (unsupervised) postgraduate general medicine placement**	306	125		*p* = *0.085*
No	269	105	1	
Yes	37	20	1.84 [0.92—3.67]	
**Rural level 1 and/or 2 postgraduate general medicine placement**	305	125		*p* = *0.067*
No	238	91	1	
Yes	67	34	1.66 [0.96—2.87]	
**Rural place of residence in the last 6 months of postgraduate placement**	300	123		*p* = *0.015*
No	287	113	1	
Yes	13	10	5.13 [1.38—19.06]	
**Locum of more than 3 months in a rural area**	328	136		*p* < *0.001*
No	231	74	1	
Yes	97	62	3.76 [2.28—6.18]	

*95%CI* 95% confidence interval

^a^During multivariate analysis, this variable had the same result and was the only significant association

Importantly, GPs with a rural upbringing were more likely to practise in a rural area (OR = 2.35; 95% CI [1.07–5.15]; *p* = 0.032) on multivariate analysis. The influence of GP place of origin on the choice of rural or urban training placements was also assessed (Table 5) but no association was revealed (*p* = 0.903 for undergraduate and *p* = 0.427 for level 1 or 2 postgraduate).

**Table 5:** Interaction results revealing no association between place of origin and rural training placements

Variable	GPs with rural upbringing	GPs with urban upbringing	*Interaction* *p-*value
**Rural undergraduate general medicine placement**	*n* = 28	*n* = 310	0.903
No	1	1	
Yes	3.71 [0.08–165.19]	2.90 [0.97–8.63]	
**Rural level 1 (supervised) postgraduate general medicine placement**	*n* = 29	*n* = 276	0.500
No	1	1	
Yes	0.99 [0.19—5.26]	1.83 [0.97—3.44]	
**Rural level 2 (unsupervised) postgraduate general medicine placement**	*n* = 29	*n* = 277	0.446
No	1	1	
Yes	0.86 [0.12—6.09]	1.94 [092—4.07]	
**Rural level 1 and/or 2 postgraduate general medicine placement**	*n* = 29	*n* = 276	0.427
No	1	1	
Yes	0.87 [018—4.15]	1.71 [0.95—3.07]	

## Discussion

To our knowledge, this is the first study to reveal that French GPs with a rural upbringing are more likely to practise in rural areas than GPs with an urban upbringing. Furthermore, significantly more spouses or partners of rural GPs had a rural upbringing and worked in a rural area. Rural locum placements lasting more than three months were also significantly associated with practising in a rural area.

### Rural upbringing

The positive association between French GPs having a rural upbringing and practising in a rural area are consistent with international literature. Canadian studies revealed that GPs who grew up in a rural area (OR = 8.37), had a rural address when they enrolled in medical school (OR = 2.61), or went to secondary school in a rural area (OR = 4.03) were significantly more likely to practise in a rural area [16–22]. Australian GPs growing up in rural areas are also more likely to become rural GPs [23, 24] and the same applies to American GPs [15].

Now that this association between a rural upbringing and practising in a rural area has been demonstrated in France, it is possible to consider applying policies successfully used in other countries which have shown this same association. Some countries, such as the USA, have chosen to select medical students based on their geographical origins in an attempt to remedy the shortage of GPs in rural areas [25]. The WHO highlighted this strategy in 2010 as a possible solution for increasing access to health workers in remote and rural areas [2]. In Australia, for instance, most medical schools are part of the RUSC programme (Rural Undergraduate Support and Coordination) in which 25% of government-funded university places are allocated to students from rural areas [26]. In France there are currently no such initiatives. This may be because selective recruitment based on positive discrimination raises ethical issues. However, promoting medical studies in rural areas and studying rural medicine during student training could be particularly beneficial [27] because, as shown in this study, most GPs have urban backgrounds, regardless of where they practise now.

### Family and personal life

In this study, most respondents were living with a partner or spouse when they started working in their current practice which is consistent with the French literature [28]. Our results reveal that spouses or partners of rural GPs are more likely to have a rural upbringing than those of urban GPs, possibly because people who have grown up in a rural area are better adapted to rural life. This concurs with an Australian study which revealed that having a partner with a rural upbringing was strongly associated with rural practise (OR = 3.14 [1.96–5.10]) [29]. Furthermore, an Australian study including more than 2000 GPs revealed that spouses or partners of rural GPs were more likely to have spent some or all of their primary schooling in a rural area [30].

Our study also reveals that the partners and spouses of rural GPs were more likely to work in rural areas than those of urban GPs. A 2019 French literature review found that the spouse’s job could be an obstacle to practising in a rural area [31]. Conversely, spouses being able to find employment supports GPs moving to rural areas [7]. This demonstrates the importance of not just focusing on the GP but also considering their family and specifically emphasising professional opportunities for spouses or partners. However, as yet, nothing has been implemented in France to support and encourage families to move to rural areas.

### Undergraduate and postgraduate training placements

This study revealed that rural GPs were more likely to have completed rural undergraduate training placements, independent of their background, which is not surprising and is consistent with existing literature [9, 18, 19]. This may be because students had already decided to practise in a rural area or are open to this possibility. Furthermore, our study reveals that where GPs were living at the end of their undergraduate training influenced where they chose to practise. This could suggest that exposure to rural living from the end of university training could influence the choice to practise in a rural area.

The influence of postgraduate placement location on practising in rural areas has already been shown [9, 18]. However, unlike existing literature, our study did not reveal a significant association between postgraduate rural placements and practising in rural areas but, the results were close to significance.

The positive association between rural training placements, particularly undergraduate, and choosing to practise in rural areas could be utilised to improve rural GP numbers. This has shown to be effective in many other countries including the USA [32], Canada [33], Japan [34], Australia [35], and Thailand [36]. In China, Guangxi Medical University established the Rural-oriented Free Tuition Medical Education (RTME) programme, and it has been shown that 100% of RTME graduates practise in rural areas compared with 1.06% of non-RTME graduates [37]. In France, policymakers seem aware of this influence. In fact, the 2019 law relating to health system organisation and transformation recommends a postgraduate outpatient medicine placement in a non-dense zone. However, the non-compulsory nature of the recommendation could limit its impact [38]. Furthermore, it has been shown that there is a cumulative effect where each week spent in non-urban placements increases the likelihood of practising there by 14% [17] meaning longer rural placements could be beneficial. This is supported by data from Jichi Medical University (JMU) in Japan whose graduates are obliged to complete a nine-year postgraduate rural placement and are four times more likely than non-JMU graduates to remain working in rural areas after this time [34]. This strategy has successfully increased the numbers and retention of rural GPs.

### Locum placements

Our study reveals a significant association between rural locum placements of at least three months and practising in a rural area. This concurs with a Canadian study showing that 44.6% of GPs choose to practise in an area where they have previously worked as a locum [39]. In France, the rate is even higher with two thirds of GPs making this choice [40]. The CGET (Commissariat Général à l'Egalité des Territoires) released a statement about the importance of professional connections when choosing where to practise [7] which supports our data. To make use of this, it could be beneficial to promote rural locum placements. Financial assistance already exists in France for this but has had little impact with only 19 recipients found in 2017 [41]. This is consistent with Australian results where the financial part of the General Practice Rural Incentive Programme only played a limited role in improving access to GPs [42]. Measures to make practising in rural areas more attractive such as multidisciplinary health centres could be proposed [7]. Coercive measures could be another option but has not been adopted in France to date.

### Strengths and limitations

The study strengths include limiting selection bias by including randomly selected controls, making it possible to compare two populations (rural and urban GPs). In addition, the study populations had similar characteristics to the general French GP population in terms of gender and age [43]. To reduce classification bias, all communes were classified according to INSEE categories to avoid subjective interpretations of what is rural.

This study does have several limitations. A restrictive definition of rural areas was used, including few GPs. It could be more relevant to use a more precise definition, differentiating into rural, semi-rural and urban areas. Furthermore, INSEE classification dating from 2010 was used corresponding to the demographic situation at that time which may have altered more or less significantly since then.

Memory bias was possible due to the retrospective nature of the study. Another bias may have come from only reminding rural GPs about the study and not urban GPs since the number of urban GPs required had already been reached. Despite this, the results are significant.

### Future perspectives

This study did not seek to determine whether GPs who had completed rural training placements and then went onto to practise in a rural area already had an interest in rural practice, or whether it was the experience that motivated them. It would be interesting to design a study to explore this parameter, as has been done in English-language countries [44, 45].

Results from this study confirm that the intrinsic and extrinsic factors associated with GPs practising in rural areas in France are similar to those found in Australia and North America. These results may therefore be of interest to other European countries, particularly those with similar health systems to France.

## Conclusion

This study revealed that French rural GPs were more likely to have grown up, trained, or worked as a locum in a rural area. Strategies to improve rural GP recruitment and retention in France could therefore include making rural areas a more attractive place to live and work, encouraging rural locum placements and compulsory rural training, and possibly enrolling more medical students with a rural background.

## Data Availability

The datasets used and analysed during the current study are available from the corresponding author on reasonable request.

## Abbreviations

GP: General Practitioner
OR: Odds Ratio
WHO: World Health Organization
RUSC: Rural Undergraduate Support and Coordination
RTME: Rural-oriented Free Tuition Medical Education
JMU: Jichi Medical University
CGET: Commissariat Général à l'Egalité des Territoires
INSEE: National Institute of Statistics and Economic Studies
